# Discovery of highly potent and selective 7-ethyl-10-hydroxycamptothecin-glucose conjugates as potential anti-colorectal cancer agents

**DOI:** 10.3389/fphar.2022.1014854

**Published:** 2022-11-23

**Authors:** Chao Yang, An-Jie Xia, Cheng-Hao Du, Ming-Xing Hu, You-Ling Gong, Rong Tian, Xin Jiang, Yong-Mei Xie

**Affiliations:** ^1^ State Key Laboratory of Biotherapy and Cancer Center, West China Hospital, Sichuan University and Collaborative Innovation Center of Biotherapy, Chengdu, Sichuan, China; ^2^ Cognitive Impairment Ward of Neurology Department, The Third Affiliated Hospital of Shenzhen University Medical College, Shenzhen, Guangdong, China; ^3^ Department of Neurology, The First Affiliated Hospital of Guangzhou Medical University, Guangzhou, Guangdong, China; ^4^ Department of Biological Sciences, USC Dana and David Dornsife College of Letters, Arts and Sciences, Los Angeles, CA, United States; ^5^ Department of Thoracic Oncology, West China Hospital, Sichuan University, Chengdu, Sichuan, China; ^6^ Department of Nuclear Medicine, West China Hospital, Sichuan University, Chengdu, Sichuan, China; ^7^ Department of Pediatric Surgery, West China Hospital, Sichuan University, Chengdu, Sichuan, China

**Keywords:** warburg effect, 7-ethyl-10-hydroxycamptothecin, glucose conjugates, colorectal cancer, anticancer

## Abstract

7-Ethyl-10-hydroxycamptothecin (SN38), a highly potent metabolite of irinotecan, has an anticancer efficacy 100–1000 folds more than irinotecan *in vitro*. However, the clinical application of SN38 has been limited due to the very narrow therapeutic window and poor water solubility. Herein, we report the SN38-glucose conjugates (Glu-SN38) that can target cancer cells due to their selective uptake *via* glucose transporters, which are overexpressed in most cancers. The *in vitro* antiproliferative activities against human cancer cell lines and normal cells of Glu-SN38 were investigated. One of the conjugates named **5b** showed high potency and selectivity against human colorectal cancer cell line HCT116. Furthermore, **5b** remarkably inhibited the growth of HCT116 *in vivo*. These results suggested that **5b** could be a promising drug candidate for treating colorectal cancer.

## Introduction

Colorectal cancer (CRC) is the third most common malignant disease and the second leading cause of cancer-related deaths worldwide (Liu et al., 2021). In 2018, there were 1.8 million new colorectal cancer cases and 881,000 deaths according to the GLOBOCAN data (Bray et al., 2018). The incidence of CRC has been steadily rising worldwide, especially in developing countries. At present, surgery combined with chemotherapy and radiotherapy still is the main treatment for CRC (Zhu et al., 2021). Recently, the survival rate of patients with CRC has improved due to the application of molecular targeted therapy and immunotherapy. However, the expensive cost limits the clinical use in patients. Therefore, exploring new strategies to treat CRC is extremely important for improving the survival rate of CRC patients.

Camptothecin (CPT), a potent topoisomerase I inhibitor, was isolated from the Asian tree Camptotheca acuminate by Wall et al. (1966). CPT plays an important role in DNA metabolism, which induces apoptosis, cell cycle progression, and other cellular responses (Heng et al., 2021). However, the poor water solubility and stability in physiological conditions and toxicity limited its clinical use (Watanabe et al., 2008). Several CPT derivatives such as irinotecan, topotecan, and 10-hydroxycamptothecin have been developed to treat lung cancer, colorectal cancer, ovarian cancer, and breast cancer. Among them, irinotecan has achieved the greatest success in clinical application and market due to its excellent efficacy. Irinotecan, the standard therapeutic agent of the first-line treatment for advanced or metastatic CRC, is bio-activated to 7-ethyl-10-hydroxy-camptothecin (SN38) by carboxylesterase *in vivo* (Si et al., 2019; Levi et al., 2022). The main side effects of irinotecan that include diarrhea and neutropenia require dose reduction or even withdrawal (Aiyangar et al., 2010; Liu et al., 2020). SN38 has 100–1000 folds more potent than irinotecan especially employed in the treatment of many malignancies such as colon cancer (Ebrahimnejad et al., 2009; Ebrahimnejad et al., 2011; Ebrahimnejad, 2016; Sharifi et al., 2021). However, the clinical application of SN38 is highly limited by some factors, for instance, poor stability or solubility (Roger et al., 2011) and lack of selectivity to tumor tissues or tumor cells, resulting in decreased therapeutic activity and severe side effects (Abigerges et al., 1995). Scientists have proposed various strategies for solving the problem of SN38, including incorporation into liposomes (Zhang et al., 2004), nanoparticles (Ebrahimnejad et al., 2010) and polymeric micelles (Gu et al., 2012), conjugating with hydrophilic molecules like hyaluronic acid (Serafino et al., 2011), polyethylene glycol (Patnaik et al., 2013), PAMAM dendrimers (Vijayalakshmi et al., 2010) *etc.* The Warburg effect is the unique characteristic of tumor cells which have a much higher demand for glucose than normal cells (Warburg, 1956; Porporato et al., 2011; Liberti and Locasale, 2016). To sustain their growth and survival, most cancer cells including colorectal, lung, breast, ovarian, and cervical cancers overexpress glucose transporters (GLUTs), especially GLUT1 and GLUT3, to increase the uptake of glucose (Carvalho et al., 2011; He et al., 2016). Therefore, the overexpression of GLUTs in tumor cells provides a new strategy for improving the selectivity of chemotherapy drugs by conjugating cytotoxic drugs to glucose (Calvaresi and Hergenrother, 2013). Inspired by this idea, glucose conjugates have been synthesized for several cytotoxic drugs such as isophosphoramide, azinomycin, adriamycin, clioquinol, cyclopamine, quinolinyl, paclitaxel, and platinum (Pohl et al., 1995; Lin et al., 2008; Goff and Thorson, 2012; Cao et al., 2013; Patra et al., 2016a; He et al., 2016). The selectivity of the drugs for tumor cell was increased, thereby reducing toxic side effects.

In this study, to enhance the water solubility, selectivity, and stability of SN38, three glucose conjugates were designed and synthesized. The physical and chemical properties, antitumor activity *in vitro* and *in vivo*, and mechanisms of action of the SN38-glucose conjugates were also investigated.

## Materials and methods

### Materials

All reagents were purchased from commercial companies and directly used unless stated otherwise. If necessary, the reactions were carried out in dry solvents and under an argon atmosphere. The human colorectal tumor cell lines HCT116, DLD1, and HT29 were purchased from ATCC. The cells were propagated in RPMI 1640 or DMEM medium containing 10% heat-inactivated fetal bovine serum (FBS; Hyclone, Logan, UT, United States) and 1% antibiotics (penicillin and streptomycin) in 5% CO_2_ at 37°C. MTT, DMSO, 2-(N-(7-Nitrobenz-2-oxa-1,3-diazol -4-yl)amino)-2-deoxyglucose (2-NBDG) and PI were from Sigma Chemical Co. (St Louis, MO, United States). The primary antibodies against Ki-67 and cleaved-caspase-3 (CC-3) were purchased from Cell Signaling Technology (Beverly, MA, United States). The Annexin V-FITC Apoptosis Detection Kit was purchased from KeyGen Biotech (Nan-jing, China). Female BALB/c nude mice (5–6 weeks old) were purchased from Beijing HFK Bioscience. Animal experiments were approved by the Institutional Animal Care and Treatment Committee of the State Key Laboratory of Biotherapy at Sichuan University.

### Chemistry

**SCHEME 1:**
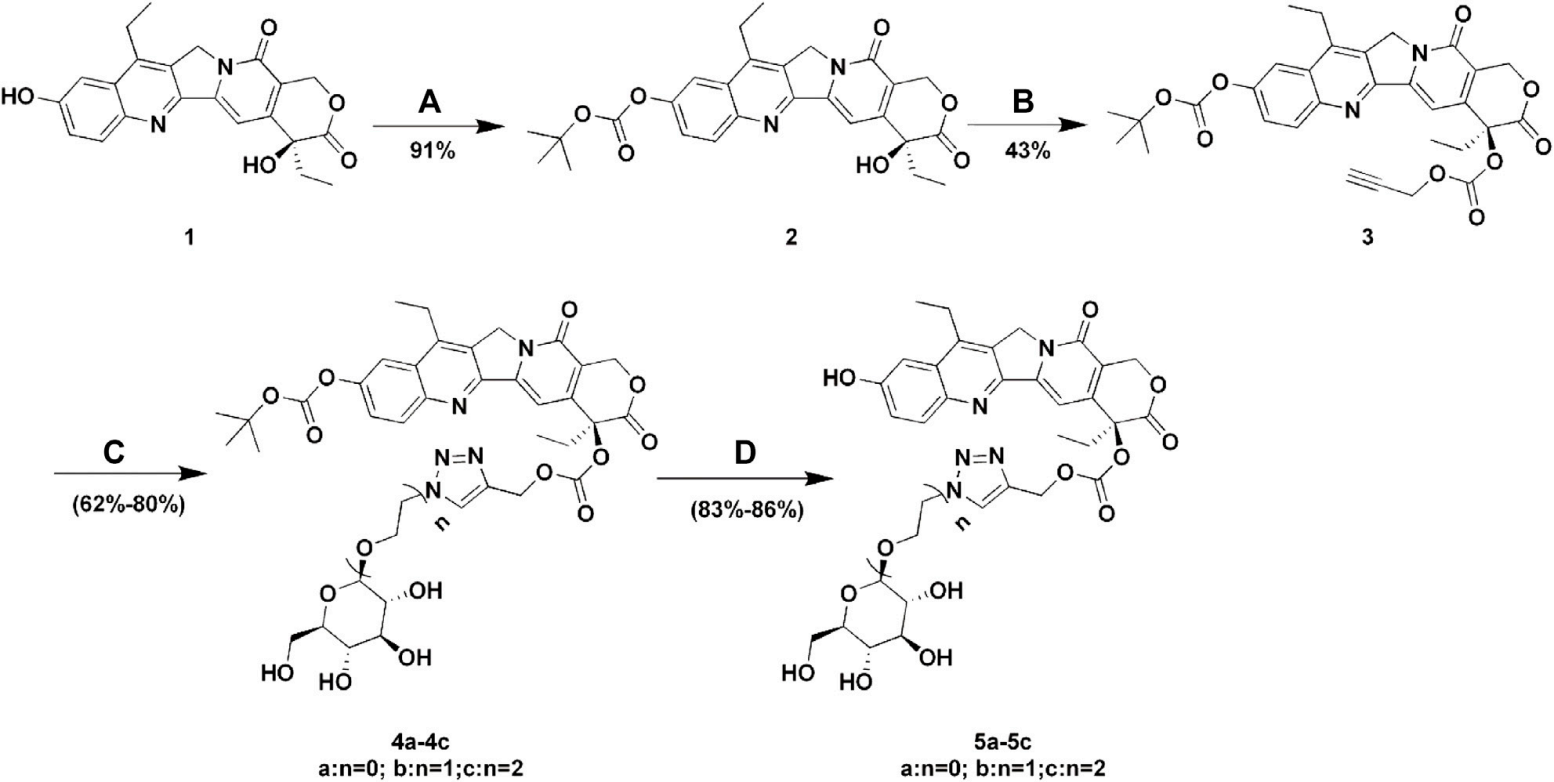
Synthesis of Glu-SN38. Reagents and conditions: **(A)** (BOC)_2_O, pyridine, DCM, 12 h; **(B)** propargyl alcohol, triphosgene, DMAP, DCM, 2 h; **(C)** glucose azides (β-D-Glucopyranosyl azide, 2-azidoethyl β-D-glucopyranoside or 2-(2-azidoethoxy) ethyl β-D-glucopyranoside), CuSO_4_^.^5H_2_O, sodium ascorbate, THF/H_2_O, 12 h; **(D)** TFA, DCM, 2 h.

SN38-glucose conjugates were synthesized according to Scheme 1. β-D-Glucopyranosyl azide, 2-azidoethyl β-D-glucopyranoside, and 2-(2-azidoethoxy)ethyl β-D-glucopyranoside were prepared as previously reported (Pintal et al., 2015; Shiao et al., 2016; Palmioli et al., 2017). ^1^H NMR and ^13^C NMR spectra were measured on a Bruker AM400 NMR spectrometer. Proton chemical shifts of NMR spectra were given in ppm relative to internals reference TMS (0.00ppm). High-resolution mass spectra were recorded on a Bruker MicroTOF spectrometer using positive (ESI^+^) or negative electrospray ionization (ESI^−^). HPLC analysis was measured on Waters 2695e equipped with a 2998 PDA detector.

### Synthesis of compound 2

To a solution of di-tert-butyl dicarbonate (361.5 mg, 1.66 mmol) and SN38 (**1**, 500 mg, 1.28 mmol) in 10 ml of anhydrous DCM were added pyridine (2 ml). The reaction mixture was stirred at 25°C overnight. After removing the solvent, DCM was added and washed with 1N HCl, saturated NaHCO_3_, and brine. The organic layer was dried over anhydrous Na_2_SO_4_, filtered, and evaporated under the vacuum. The residue was purified by flash column chromatography on silica gel (DCM: MeOH = 100: 1) to give compound **2** (579 mg, 91%).

^1^H NMR (400 MHz, DMSO-*d*_*6*_) δ 8.20 (d, *J* = 9.2 Hz, 1H), 8.09 (d, *J* = 2.5 Hz, 1H), 7.73 (dd, *J* = 9.2, 2.5 Hz, 1H), 7.33 (s, 1H), 6.52 (s, 1H), 5.44 (s, 2H), 5.33 (s, 2H), 3.20 (q, *J* = 7.6 Hz, 2H), 1.88 (m, 2H), 1.54 (s, 9H), 1.29 (t, *J* = 7.6 Hz, 3H), 0.89 (t, *J* = 7.3 Hz, 3H).

### Synthesis of compound 3

To a solution of compound **2** (500 mg, 1.02 mmol) and triphosgene (112 mg, 0.38 mmol) in 10 ml of anhydrous DCM were added DMAP (397 mg, 3.25 mmol). After stirring at 25°C for 0.5 h, propargyl alcohol (0.3 ml, 5 mmol) was added and further stirred for 1 h, then the reaction mixture was washed with water and brine. The organic layer was dried over anhydrous Na_2_SO_4_, filtered, and evaporated under the vacuum. The residue was purified by flash column chromatography on silica gel (EA: PE = 1: 1) to give compound **3** (252 mg, 43%).

^1^H NMR (400 MHz, CDCl_3_) δ 8.23 (d, *J* = 9.2 Hz, 1H), 7.90 (d, *J* = 2.5 Hz, 1H), 7.68 (dd, *J* = 9.2, 2.5 Hz, 1H), 7.29 (s, 1H), 5.71 (d, *J* = 17.2 Hz, 1H), 5.39 (d, *J* = 17.2 Hz, 1H), 5.25 (d, *J* = 2.3 Hz, 2H), 4.70 (t, *J* = 2.6 Hz, 2H), 3.16 (q, *J* = 7.7 Hz, 2H), 2.51 (t, *J* = 2.4 Hz, 1H), 2.36–2.07 (m, 2H), 1.62 (s, 9H), 1.39 (t, *J* = 7.7 Hz, 3H), 1.01 (t, *J* = 7.5 Hz, 3H). ^13^C NMR (100 MHz, CDCl_3_) δ 167.06, 157.28, 153.10, 151.74, 151.49, 150.00, 147.31, 146.98, 145.47, 131.89, 127.49, 127.18, 125.31, 120.19, 114.22, 99.99, 95.87, 84.45, 78.33, 76.50, 76.19, 67.06, 56.18, 49.34, 31.87, 27.73, 27.73, 27.73, 23.21, 13.97, and 7.63.

### Synthesis of compound 4a

To a solution of **3** (100 mg, 0.17 mmol) and β-D-glucopyranosyl azide (40 mg, 0.20 mmol) in THF/H_2_O (5 ml/5 ml) were added sodium ascorbate (99 mg, 0.50 mmol) and CuSO_4_^.^5H_2_O (75 mg, 0.3 mmol). The resulting mixture was stirred overnight at room temperature and was directly purified by column chromatography on silica gel (DCM: MeOH = 20:1) to afford **4a** (218 mg, 80%).

^1^H NMR (400 MHz, DMSO-*d*_6_) δ 8.43 (s, 1H), 8.25 (d, *J* = 9.2 Hz, 1H), 8.14–7.99 (m, 1H), 7.85–7.65 (m, 1H), 7.07 (s, 1H), 5.56 (d, *J* = 6.9 Hz, 3H), 5.47–5.10 (m, 7H), 4.63 (t, *J* = 5.6 Hz, 1H), 3.71 (m, 2H), 3.44 (m, 3H), 3.23 (m, 3H), 2.27–2.07 (m, 2H), 1.55 (s, 9H), 1.29 (t, *J* = 7.6 Hz, 3H), 0.91 (t, *J* = 7.3 Hz, 3H). ^13^C NMR (100 MHz, DMSO-*d*_6_) δ 167.51, 156.92, 153.18, 152.16, 151.52, 149.82, 147.13, 146.89, 146.87, 140.08, 140.08, 131.82, 129.07, 127.59, 125.11, 125.80, 119.72, 115.40, 94.77, 88.00, 84.26, 80.43, 78.37, 77.36, 72.61, 69.96, 67.00, 61.86, 61.16, 50.12, 30.90, 27.77, 27.77, 27.77, 22.71, 14.31, and 7.98.

### Synthesis of compound 5a

Compound **4a** (50 mg, 0.06 mmol) was dissolved in 20 ml of 30% TFA in DCM. The mixture was stirred for 2 h, and then the solvent was removed under the vacuum. The residue was purified by flash column chromatography on silica gel (DCM: MeOH = 20: 1) to give compound **5a** (35 mg, 86%).

^1^H NMR (400 MHz, DMSO-*d*_6_) δ 10.43 (s, 1H), 8.43 (s, 1H), 8.06 (d, *J* = 9.9 Hz, 1H), 7.47–7.38 (m, 2H), 6.97 (s, 1H), 5.58–5.48 (m, 3H), 5.42 (d, *J* = 5.9 Hz, 1H), 5.36–5.13 (m, 6H), 4.64 (t, *J* = 5.7 Hz, 1H), 3.80–3.59 (m, 2H), 3.48–3.38 (m, 3H), 3.24 (m, 1H), 3.09 (q, *J* = 7.5 Hz, 2H), 2.17 (m, 2H), 1.30 (t, *J* = 7.5 Hz, 3H), 0.91 (t, *J* = 7.4 Hz, 3H). ^13^C NMR (100 MHz, DMSO-*d*_6_) δ 167.59, 157.38, 157.00, 153.15, 148.92, 147.74, 145.18, 144.08, 143.36, 140.88, 132.02, 128.79, 128.47, 125.10, 123.01, 118.66, 105.28, 93.84, 88.00, 80.43, 78.42, 77.35, 72.61, 69.96, 66.98, 61.84, 61.17, 50.05, 30.86, 22.77, 13.82, 7.97. HRMS (ESI, m/z) calfd. for C_32_H_33_N_5_O_12_ (M + Na)^+^ 702.2023, found 702.2040.

### Synthesis of compound 5b

Compound **5b** was prepared according to the procedure described for compound **5a**, starting from 2-azidoethyl β-D-glucopyranoside and **3**.

^1^H NMR (400 MHz, DMSO-*d*_6_) δ 10.37 (s, 1H), 8.25 (s, 1H), 8.05 (d, *J* = 8.9 Hz, 1H), 7.49–7.36 (m, 2H), 6.93 (s, 1H), 5.52 (s, 2H), 5.36–5.14 (m, 4H), 5.05 (d, *J* = 4.9 Hz, 1H), 4.94 (dd, *J* = 10.6, 4.9 Hz, 2H), 4.61–4.38 (m, 3H), 4.21 (d, *J* = 7.8 Hz, 1H), 4.02 (ddd, *J* = 10.7, 6.0, 4.4 Hz, 1H), 3.86 (ddd, *J* = 11.0, 6.3, 4.4 Hz, 1H), 3.67 (ddd, *J* = 11.8, 6.0, 2.0 Hz, 1H), 3.44 (m, 1H), 3.20–2.87 (m, 6H), 2.16 (m, 2H), 1.30 (t, *J* = 7.5 Hz, 3H), 0.91 (t, *J* = 7.4 Hz, 3H). ^13^C NMR (100 MHz, DMSO-*d*_6_) δ 167.59, 157.34, 157.00, 153.14, 148.94, 147.69, 145.23, 143.36, 140.74, 132.02, 128.76, 128.46, 126.60, 123.02, 118.65, 105.27, 103.35, 93.82, 78.40, 77.44, 77.03, 73.73,70.44, 67.67, 66.95, 61.91, 61.53, 55.36, 50.17, 50.03, 30.82, 22.77, 13.84, and 7.98. HRMS (ESI, m/z) calfd. for C_34_H_37_N_5_O_13_ (M + Na)^+^ 746.2286, found 746.2291.

### Synthesis of compound 5c

Compound **5c** was prepared according to the procedure described for compound **5a**, starting from 2-(2-azidoethoxy) ethyl β-D-glucopyranoside and **3**.

^1^H NMR (400 MHz, DMSO-*d*_6_) δ 10.42 (s, 1H), 8.17 (s, 1H), 8.05 (d, *J* = 8.9 Hz, 1H), 7.43 (d, *J* = 9.1 Hz, 2H), 6.93 (s, 1H), 5.52 (s, 2H), 5.25 (m, 4H), 5.08–4.83 (m, 3H), 4.57–4.35 (m, 3H), 4.14 (d, *J* = 7.8 Hz, 1H), 3.87–3.40 (m, 8H), 3.19–2.93 (m, 6H), 2.26–2.05 (m, 2H), 1.30 (t, *J* = 7.5 Hz, 3H), 0.91 (t, *J* = 7.3 Hz, 3H).

^13^C NMR (100 MHz, DMSO-*d*_6_) δ 167.59, 157.38, 157.00, 153.17, 148.92, 147.67, 145.25, 144.06, 143.36, 140.91, 131.99, 128.77, 128.45, 126.14, 123.02, 118.63, 105.28, 103.39, 93.85, 78.40, 77.33, 77.19, 73.86, 70.52, 69.88, 69.05, 68.20, 66.94, 61.91, 61.53, 50.03, 49.82, 30.82, 22.77, 13.83, and 7.98. HRMS (ESI, m/z) calfd. for C_36_H_41_N_5_O_14_ (M + Na)^+^ 790.2548, found 790.2585.

### Determination of octanol-water partition coefficient (log *P*)

The oil-water partition coefficient values of the compounds were determined by the shake-flask method. Octanol used in this experiment was pre-saturated with distilled water by overnight incubation with shaking of a biphasic mixture of the two at rt. A portion of 0.5 ml pre-saturated octanol containing 10 μg/ml of analyte was incubated with distilled water (0.5 ml) in a 1.5 ml tube. The tube was covered with aluminum foil and was shaken at 37°C for 3 h using an automatic shaker. Two phases were then separated by centrifugation and the compound content in each phase was determined by HPLC. All the experiments were carried out in triplicate.

### Cell viability assay

The cells were seeded in 96-well plates and incubated in a 5% CO_2_ atmosphere in 100 µL of the complete medium at 37°C for 12 h. Then 100 µL of freshly prepared culture mediums containing drugs at different concentrations were added and incubated for another 72 h. MTT (5 mg/ml, 20 µL) was added and incubated for 4 h. Finally, the medium was removed and DMSO (150 µL) was added. The absorbance was measured at 570 nm using a Bio-Rad 680 microplate reader. The IC_50_ values were calculated using GraphPad Prism seven software, which were based on three parallel experiments.

For cytotoxicity assays using a 10 h incubation, a similar procedure was followed except the compound containing medium was replaced with fresh medium (200 µL/well) and incubated for an additional 62 h before the cell viability was determined by the MTT assay.

### 2-NBDG-mediated transportability assay

Cells were plated in 6-well plates at 1 × 10^6^ cells per well and allowed to adhere overnight. After the medium was removed, the cells were incubated for 6 h in glucose-free RPMI-1640. Following the addition of test compounds (40 µM) for 40 min, the cells were treated with 2-NBDG (80 µM) for additional 30 min. The cells were then washed with PBS (1 ml), detached by addition of Trypsin-EDTA (400 µL), and incubated for 3 min at 37°C. Following the addition of RPMI-1640 (1 ml), the cell suspensions were centrifuged at 125 g for 10 min. The resulting pellets were suspended in PBS (0.5 ml) and transferred to a flow cytometry tube. Finally, the cells were stored on ice and analyzed by flow cytometry within 1 h. Data were collected as the average of three sets of geometric means of the flow cytometry histogram and plotted as the percent difference from the control plus/minus the standard deviation between three values.

### Apoptotic assay

For cell apoptosis, the HCT116 cells (1 × 10^5^ cells/well) incubated in 6-well plates were treated with solvent control (DMSO), and various concentrations of compounds for 24 h. After 24 h incubation, all the cells were harvested. After double staining with fluorescein isothiocyanate (FITC)-conjugated Annexin V and propidium iodide (PI), the cells were analyzed by flow cytometer (FACS Canto II, BD).

### Wound-healing assay

The HCT116 cells (1 × 10^5^ cells/well) were cultured in 6-well plates until 95% confluence was reached. A single wound was then scratched in the center of the cell monolayers with a 200 μL sterile plastic pipette tip. The wounded monolayers were washed twice to remove the non-adherent cells and were incubated with various concentrations of the compound. To measure the length of the endothelial cells that had migrated from the edge of the injured monolayer, images were obtained immediately after wounding and after a 24 h incubation period, using a phase-contrast microscope.

### Stability assay

90 µL of pre-warmed plasma or PBS buffer pH 5.50, pH 7.40 was added into the wells designated for all the time points (0, 5, 15, 30, 60, and 120 min). 400 µL of ACN containing internal standard (Tolbutamide) was added to the wells of 0 min plate and then added 10 µL of pre-warmed 0.01 mM ACN solution of **5b**. For other time points, added 10 µL of pre-warmed 0.01 mM ACN solution of **5b** into the wells designated for the time points (5, 15, 30, 60, and 120 min), and started timing. At 5, 15, 30, 60, and 120 min, added 400 µL of ACN containing internal standard to the wells of corresponding plates, respectively, to stop the incubation. After quenching, shakeed the plates for 5 min (600 rpm) and stored at −20°C until analysis by LC/MS/MS. Before LC/MS/MS analysis, thawed the samples at RT and centrifuged at 12000 rpm for 10 min. Transfer 120 μL of the supernatant from each well into a 96-well sample plate containing 120 μL of water for LC/MS/MS analysis. The MS/MS was performed on SCIEX Triple Quad™ 5500 + in ESI mode. All data were calculated and plotted using GraphPad Prism 5.

### *In vivo* antitumor evaluation

To develop the human tumor xenografts, HCT116 cells were harvested. After being washed with serum-free RPMI-1640 medium, the cell suspension (1 × 10^7^, 100 µL) was implanted into the right flanks of BALB/c nude mice. When the tumor node reached 100 mm^3^, mice were randomly divided into three groups (*n* = 6): control group (normal saline), high dosage group (20 mg/kg), and low dosage group (10 mg/kg). **5b** (200 µL) and normal saline (200 µL) were injected by tail vein into mice at 1, 5, and 9 days. The tumor volume and body weight were monitored every 2 days. The tumor volume (mm^3^) was calculated using the following formula: V (mm^3^) = A (mm) × B (mm)^2^/2, where A and B were the longest and widest diameter of the tumor, respectively. When the tumor volume was over 1000 mm^3^, animals were sacrificed according to institutional guidelines at the end of the experiments. Blood routine analysis and blood chemistry analysis were performed. The tissues of the heart, liver, spleen, lung, and kidney were stained with H&E for histopathologic examination.

### Immunohistochemistry determination of Ki-67 and CC-3

The tumor tissues were fixed with 4% paraformaldehyde, dehydrated, embedded, and sliced. The expression of cell proliferation-related protein Ki67 and apoptosis-related protein CC-3 were stained with Ki-67 and CC-3 antibodies. Images were taken with the Leica microscope (Leica, DM4000B).

### Statistical analysis

Data were represented as the mean ± standard deviation (SD). The statistical comparisons were made by Student’s t-test. Statistically significant *p* values were marked as follows: *p < 0.05; **p < 0.01; ***p < 0.001.

## Results and discussion

### Chemistry

The lactone ring of SN38 is easily hydrolyzed under physiological conditions to form a carboxylate, which is the key reason for drug inactivation. It has been reported that the chemical modification of the 20-position hydroxyl group achieved an improvement in drug stability (Moon et al., 2008). In addition, the linker length leads to different spatial barriers in the process of drug-protein binding, which in turn affects drug uptake efficiency (Patra et al., 2016b). For the reasons mentioned above, we designed and synthesized three SN38-glucose conjugates (**5a-5c**) according to Scheme 1. The reaction conditions of these operations were mild, and the reactions were easy to carry out. All final products were characterized by ^1^H NMR, ^13^C NMR, and HRMS. The purity of the compounds was determined by HPLC.

### Octanol-water partition coefficient (log *P*)

Octanol-water partition coefficient (log *p*) is frequently used as a measure of lipophilicity in drug discovery. As summarized in Table 1, the log *p* of the glucose conjugates is significantly lower than that of SN38. Furthermore, as the linker length increases, the log *p*-value decreases. These results implied that glucose could improve the hydrophilicity of SN38.

**TABLE 1:** Octanol-water partition coefficient of compounds.

Compounds	Log p
**5a**	0.48 ± 0.02
**5b**	0.17 ± 0.01
**5c**	−0.06 ± 0.01
**SN38**	1.70 ± 0.02

### Antiproliferative activity assay

The cytotoxicity of **5a-5c** against three human colorectal cancer cell lines was evaluated by MTT assay and compared to that of irinotecan. As shown in Table 2, after 72 h incubation, **5a-5c** exhibited superior anti-tumor activity to irinotecan in colorectal cancer cells, especially in HCT116 cells. The cytotoxicity of **5a-5c** on HCT116 cells was about 124 times higher than that of irinotecan. There was no significant difference in the antitumor activity of compounds **5a, 5b,** and **5c**.

**TABLE 2:** Antiproliferative activity of compounds **5a, 5b,** and **5c**.

Cell lines	IC_50_ (μM)			
	5a	5b	5c	Irinotecan
HCT116	0.042 ± 0.04	0.045 ± 0.02	0.044 ± 0.02	5.15 ± 1.3
DLD1	6.08 ± 0.34	6.66 ± 0.42	6.16 ± 2.48	30.29 ± 3
HT29	2.18 ± 0.17	1.95 ± 0.07	2.05 ± 0.26	42.70 ± 2.01

### 2-NBDG-mediated transportability assay

**FIGURE 1:**
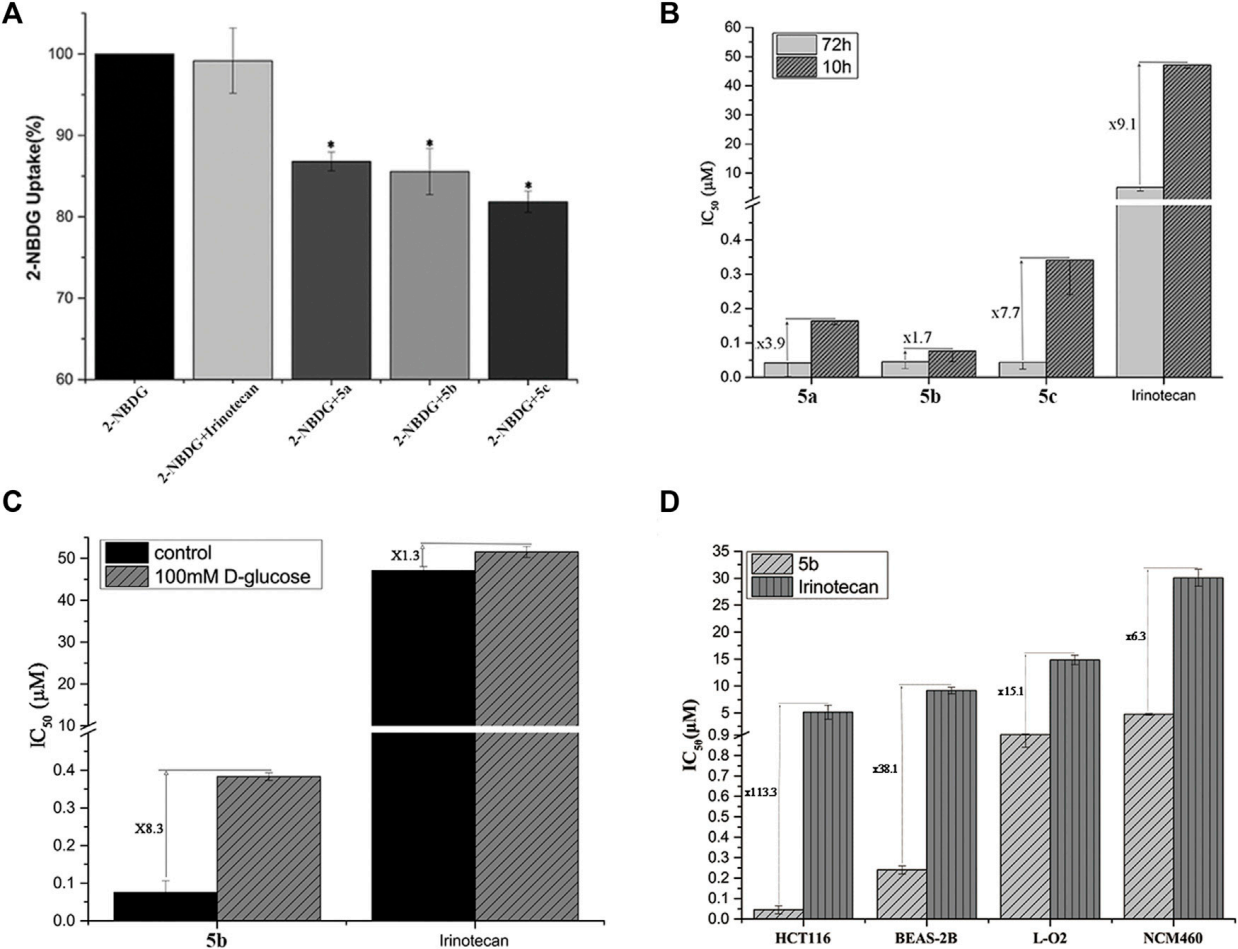
**(A)** Uptake of Glu-SN38 *via* glucose transporters using fluorescent probe 2-NBDG as competitor in HCT116 cells. **(B)** Effect of incubation time on IC_50_ values. **(C)** Effect of D-glucose on IC_50_ values. **(D)** Changes in IC_50_ values of **5b** and irinotecan in cancer and normal cells.

2-NBDG, a fluorescent tracer, has been widely used to assess GLUT-mediated glucose uptake (Hassanein et al., 2011). In this assay, the addition of glycosyl compounds that compete with 2-NBDG for binding to GLUTs resulted in a decrease in intracellular fluorescence emission detected by flow cytometry. The most sensitive HCT116 cells were used to perform competitive 2-NBDG cell uptake. The data were presented as a percentage relative to control cells with only 2-NBDG added. As shown in Figure 1A, the uptake of 2-NBDG by cells was significantly reduced when the medium contained glucose conjugates. In contrast, the introduction in the cell growth media of irinotecan, which has no glucose targeting unit, did not affect the uptake of 2-NBDG by cells. It is worth noting that with the extension of the linker coupled glucose and SN38, the effect of conjugates on cellular uptake was also increased. This result may be caused by the space barrier of the glucose conjugates and protein.

According to the antiproliferative activity assay, different linkers do not seem to cause significant cytotoxicity differences after 72 h of incubation. We therefore treated HCT116 cells with **5a-5c** for 10 h instead of 72 h, followed by 62 h incubation in fresh media. As shown in Figure 1B, the IC_50_ value of irinotecan increased by 9.1 times, while the IC_50_ value of **5a-5c** changed 3.9, 1.7, and 7.7 times, respectively, which further indicated the importance of the linker.

To further validate the targeting of glucose conjugates to glucose transporters, we performed another competitive assay using D-glucose. The most active **5b** and irinotecan were selected for the experiment. When 100 mM glucose was added to the medium, the IC_50_ values of **5b** and irinotecan changed 8.3 and 1.3 times, respectively (Figure 1C). This also confirms that **5b** could indeed enter cells by targeting glucose transporters.

An important purpose of introducing a glucose moiety is to increase the selectivity of chemotherapeutic drugs to tumor cells, thereby reducing their side effects. Human normal lung epithelial cells (BEAS-2B), intestine epithelium cells (NCM460) and liver cells (LO2) were selected to assess the selectivity of **5b** and irinotecan as a positive control. As shown in Figure 1D, HCT116 cells have a higher selectivity for **5b** than normal cells.

### Compound 5b induced cells apoptosis

**FIGURE 2:**
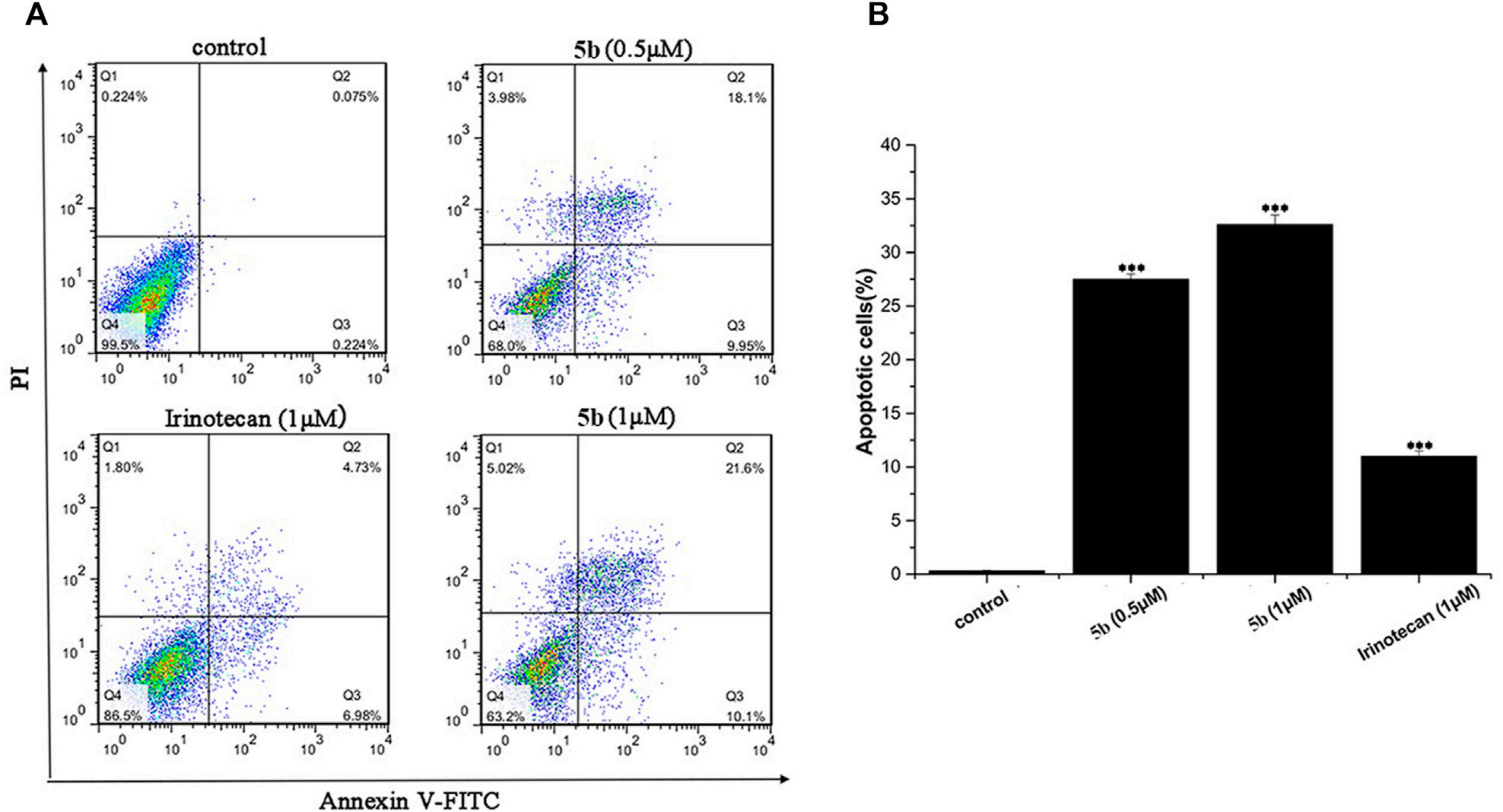
**5b** induced HCT116 cells apoptosis **(A)**The apoptosis rate of HCT116 cells treated by **5b** and irinotecan. **(B)** Statistic results of apoptosis assays. Data are expressed as mean ± S.D. from three independent experiments (***p < 0.001).

We used flow cytometry analysis to examine the effects of **5b** on HCT116 cells apoptosis. As shown in Figure 2, after incubation for 24 h, the apoptosis induction effects were observed. When the HCT116 cells were treated with 0.5 and 1.0 μM of **5b**, the apoptosis rate was 28.1% and 31.7%, respectively, indicating that **5b** was able to induce apoptosis in a concentration-dependent manner. However, the apoptosis rate of irinotecan was only 11.7% at 1.0 μM. Collectively, these results showed that **5b** has a significantly stronger ability to induce apoptosis than the apoptosis ability of irinotecan.

### Compound 5b impaired HCT116 cells migration

**FIGURE 3:**
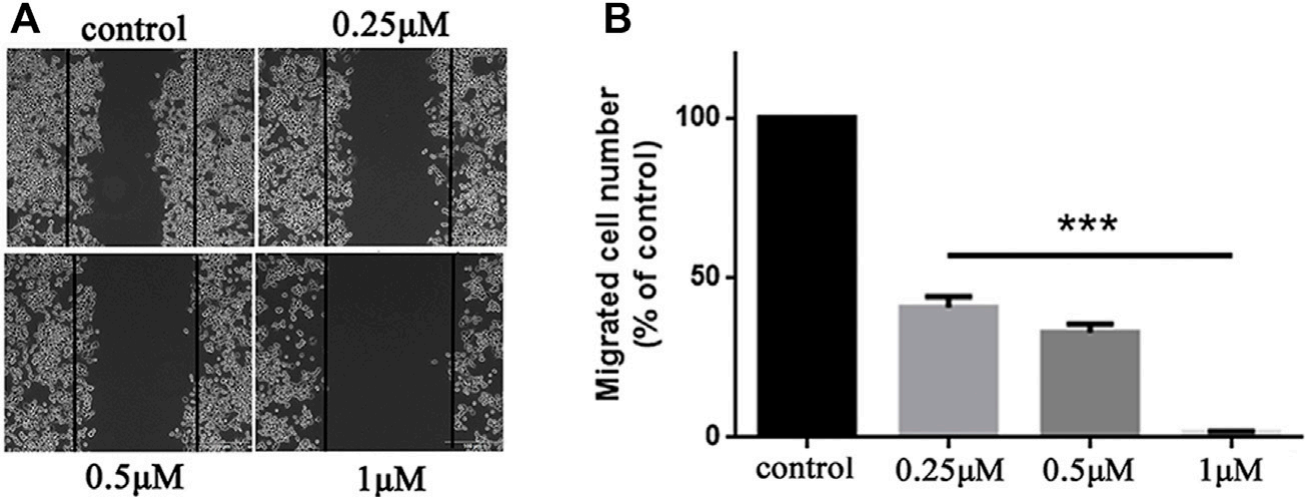
**5b** inhibited HCT116 cells migration. **(A)** Wound healing assay. **(B)** Statistic results of wound healing assay (***p < 0.001).

Metastasis is a major obstacle to anticancer therapies and is responsible for most therapeutic failures. A wound healing assay was performed to explore the effects of **5b** on HCT116 cells migration. As shown in Figure 3, **5b** reduced the wound healing ability of HCT116 cells in a dose-dependent manner. The width of the wounds in the control was significantly smaller than in the concentration of 0.25, 0.5, and 1 μM. This result implied that **5b** strongly inhibited HCT116 cells migration.

### Stability assay

**FIGURE 4:**
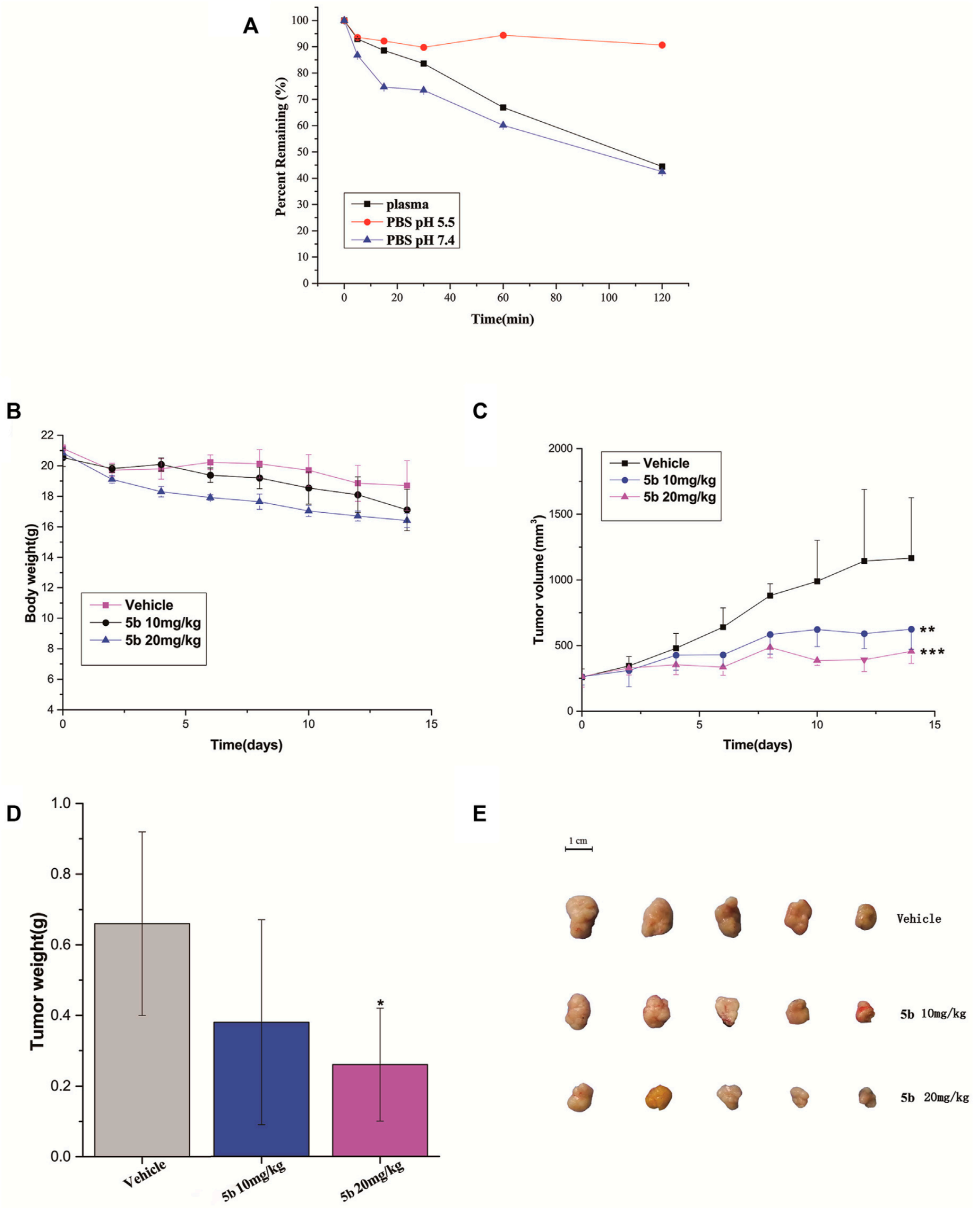
**(A)** Stability of compound 5b in PBS (pH 5.5 and pH 7.4) and plasma. **(B)** The body weight of the mice during the treatment. **(C)** Tumor growth of HCT116 model inhibited by **5b** (**p < 0.01; ***p < 0.001). **(D)** Represented weight of tumors from mice of different groups, respectively. Data were mean ± S.D. (*n* = 5, *p < 0.05). **(E)** Representative photographs of subcutaneous tumors in the HCT116 model.

We firstly measured the release profiles of compound **5b** in PBS (pH 5.5 and pH 7.4) and plasma with LC-MS/MS. As can be seen from Figure 4A, **5b** showed suitable biostability in plasma, normal pH (7.4) PBS, and PBS mimicking the tumor microenvironment at low pH (5.5), with predicted half-lives of approximately 105, 107, and >120 min, respectively. Therefore, we believe that **5b** is sufficiently stable in the circulatory system to ensure that it reaches the tumor site in an inactivated state.

### *In vivo* antitumor activity

The *in vivo* anticancer activity of **5b** was examined in the HCT116 tumor xenograft BALB/c nude mice model. The mice were injected with **5b** once every 3 days at the dose of 10 and 20 mg/kg three times. The body weight and tumor volume were measured every 2 days during the treatments. As can be seen from Figure 4B, there were no significant changes in body weight in normal saline and **5b** treated mice. Figures 4C–E shows the tumor volume curves, tumor weight as well as the tumor photos of each group. The results demonstrated that **5b** has strong antitumor activity *in vivo*.

**FIGURE 5:**
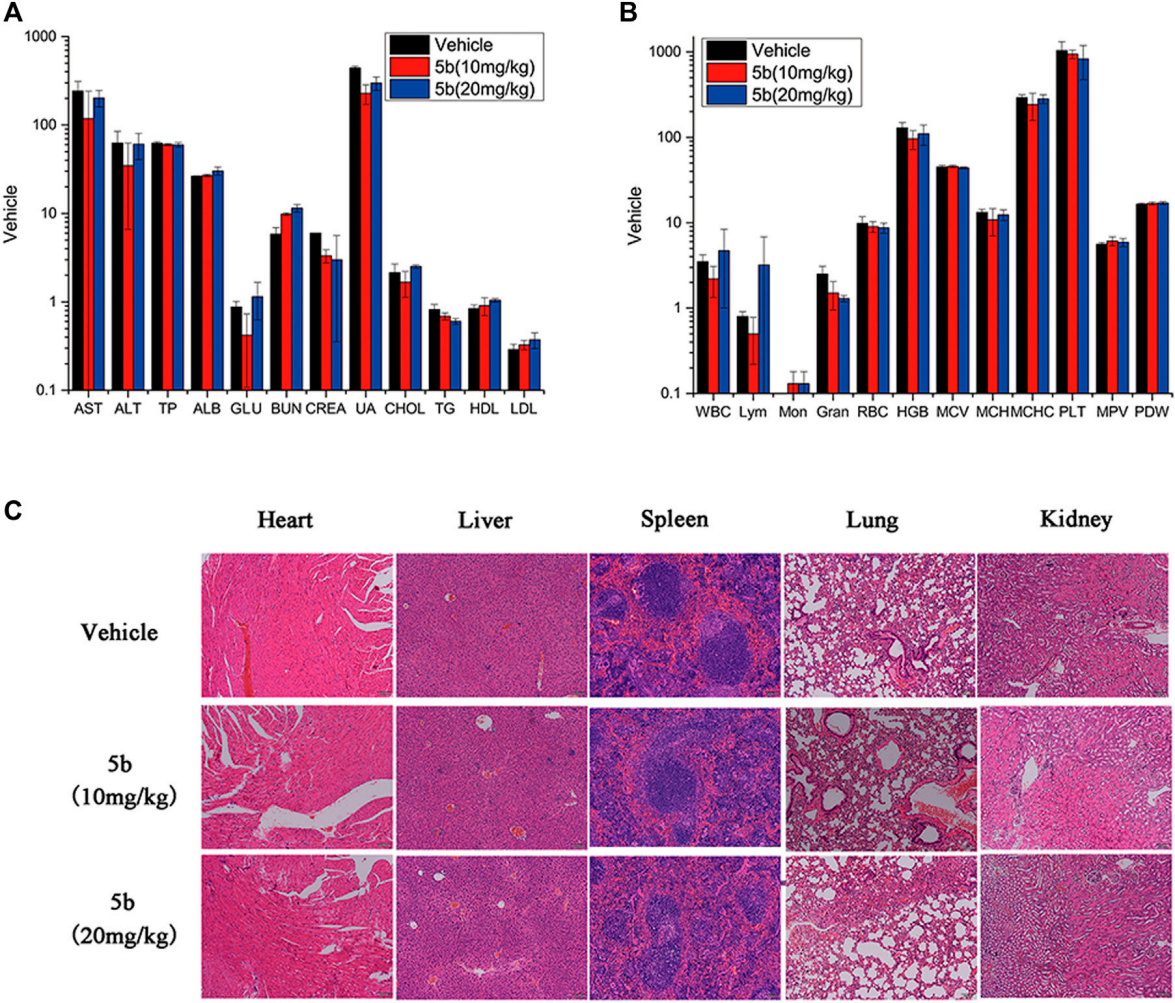
Evaluation of side effects of **5b** in mice. **(A)** Blood chemistry analysis. **(B)** Blood routine analysis. **(C)** HE staining of the sections of the liver, kidney, spleen, lung, and heart from the mice in four groups after treatment.

As mentioned above, during the treatment of HCT116 tumor-bearing mice, no obvious body weight change was observed. And there was no vomiting, toxic death, or skin ulceration in the **5b**-treated groups. To further explore the safety profile of **5b,** blood routine analysis, blood chemistry analysis, and the H&E of main organs were investigated. From Figures 5A,B, there were no significant differences in blood biochemical indicators and blood routine indicators between the treated groups and the vehicle group. Moreover, in Figure 5C, no distinct pathological morphologic change was observed after the **5b** therapy occurred in the heart, liver, spleen, lung, and kidney compared with the vehicle group. These results suggest that compound **5b** has a good safety profile at doses administered.

### Immunohistochemical analysis

**FIGURE 6:**
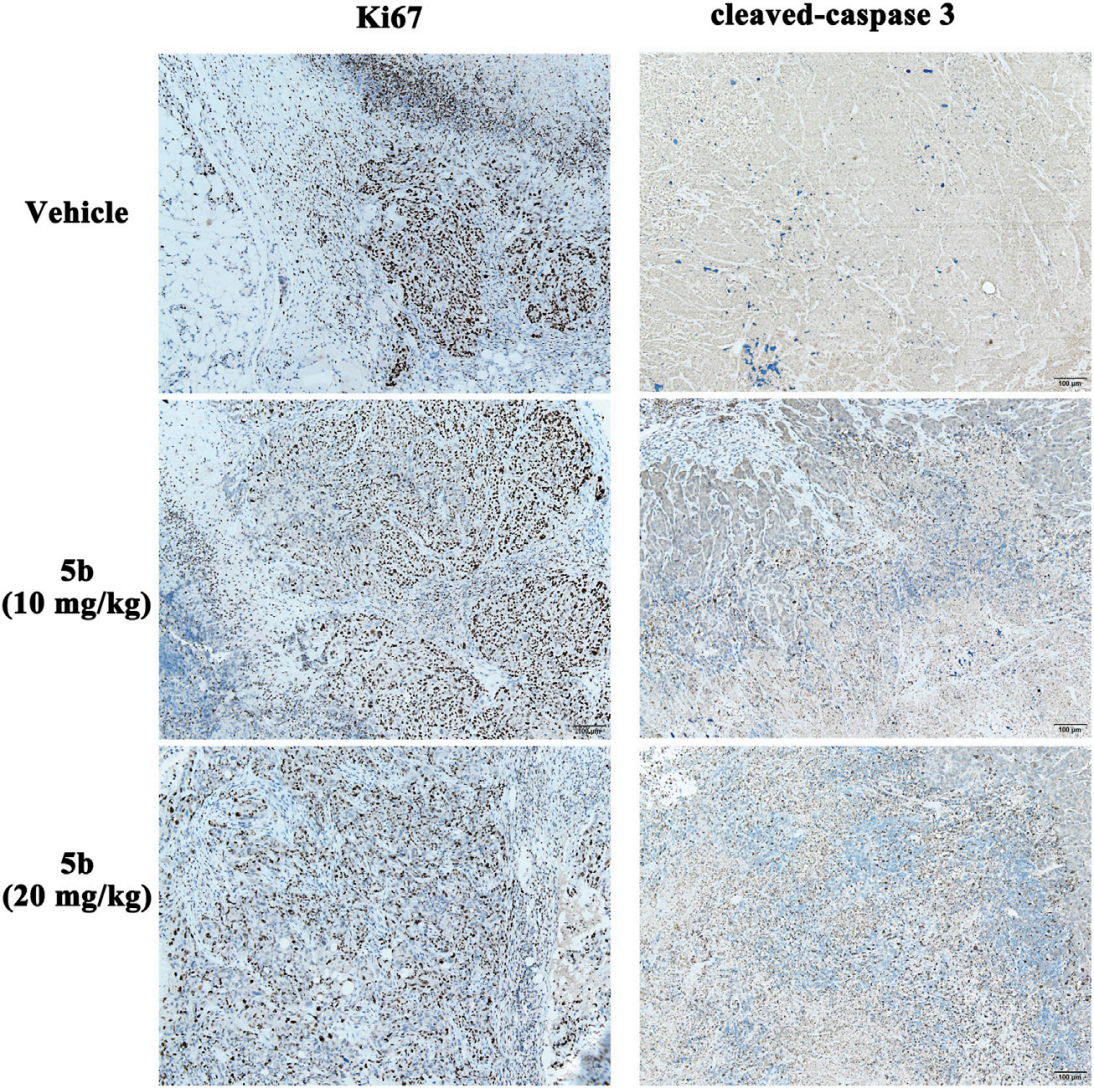
Expression of Ki-67 and CC3 in HCT116 tumor tissues.

In the *in vitro* study, we found that **5b** could inhibit cell proliferation and induce apoptosis in HCT116 cells. Ki-67 and cleaved caspase-3 (CC3) are excellent markers of proliferating and apoptotic cells, respectively (Ogane et al., 2018). To detect the effect of **5b** on Ki-67 and CC3 in the tumor tissues, immunohistochemical analysis was performed using anti-Ki-67 and anti-CC3 monoclonal antibodies. As can be seen from Figure 6, the expression of Ki-67 in the **5b** treated groups was significantly lower than that in the vehicle group. Moreover, compared with the vehicle group, CC-3-positive cells were apparently increased in the **5b** treated groups.

## Conclusion

In summary, we designed and synthesized three conjugates of glucose and SN38, and evaluated their anti-tumor activity against three colorectal cancer cell lines *in vitro* and *in vivo*. Among these compounds, **5b** demonstrated potent cytotoxicity and selectivity against HCT116 cells. **5b** could effectively inhibit tumor cell proliferation and induce cell apoptosis. Furthermore, **5b** remarkably inhibited the growth of HCT116 *in vivo*. These results suggested that **5b** could be a promising drug candidate for treating colorectal cancer.

## Data Availability

The original contributions presented in the study are included in the article/Supplementary Material, further inquiries can be directed to the corresponding authors.
